# Invasive Colonic Mucormycosis in an Immunocompromised Postliver Transplant Patient

**DOI:** 10.14309/crj.0000000000000450

**Published:** 2020-08-20

**Authors:** Pankaj Aggarwal, Harsh Patel, Lisandro Gonzalez, Landon Brown, Apeksha Agarwal, Kermit Speeg

**Affiliations:** 1 Department of Internal Medicine, University of Texas Health Science Center San Antonio, San Antonio, TX; 2 Department of Gastroenterology and Hepatology, University of Texas Health Science Center San Antonio, San Antonio, TX; 3 Department of Pathology, University of Texas Health Science Center San Antonio, San Antonio, TX

## Abstract

Mucormycosis (zygomycosis) is an invasive fungal disease caused by *Rhizopus* species, most commonly implicated in diabetic ketoacidosis and other immunocompromised states. This report presents a unique case of colonic mucormycosis in a patient several weeks after liver transplant. The patient's course was complicated by polymicrobial infection and intra-abdominal abscesses, ultimately leading to septic shock and death. This case is the first of its kind to describe such malignant abdominal mucormycosis in a solid organ transplant recipient.

## INTRODUCTION

Mucormycosis is an invasive fungal infection caused by *Rhizopus* species, most classically seen in diabetic ketoacidosis, which typically causes rhino-orbital disease but can also cause significant pulmonary disease.^1^ *Rhizopus* spores are inhaled, transported to the pharynx, and eventually cleared by the gastrointestinal tract.^2^ However, intravenous catheters, burn wounds, pressure dressings, and even minor breaks in the skin have been implicated as potential sources of entry for this pathogen.^1^ In addition to patients with diabetes mellitus, hematopoietic and solid organ transplant recipients are at a significant risk of infection with *Rhizopus.* Although the exact figures are unknown, incidence of invasive mucormycosis is estimated to be less than 5% in solid organ transplant recipients.^3^ This case describes a unique case of invasive gastrointestinal mucormycosis causing septic shock after liver transplant.

## CASE REPORT

**Figure 1.**
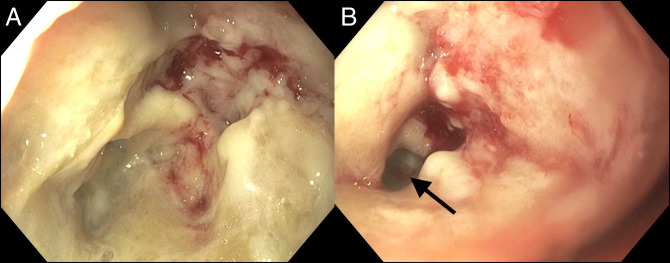
Colonoscopy showing (A) cratered, ulcerative mass with significant overlying fibrinous exudate in the sigmoid colon approximately 25 cm from the anal verge and (B) highly friable mucosa with significant narrowing of the lumen (arrow).

**Figure 2.**
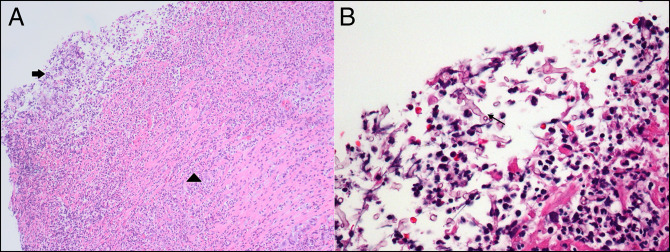
(A) Low-power view of sigmoid colon biopsy showing ulceration (arrow) with fibrinopurulent exudate and marked acute inflammation (arrowhead) (Hematoxylin and Eosin stain, magnification 100×). (B) High-power view of sigmoid colon biopsy showing pauci-septate, broad fungal hyphae (arrow) in the fibrinopurulent exudate (Hematoxylin and Eosin stain, magnification 400×).

A 70-year-old-man with cirrhosis secondary to nonalcoholic steatohepatitis complicated by hepatocellular carcinoma presented for an orthotopic liver transplant with a model for end-stage liver disease score of 12. After transplant, the patient was started on tacrolimus, mycophenolate mofetil, and methylprednisolone for immunosuppression. No T-cell depleting agents nor interleukin receptor antagonists were administered in the induction of his immunosuppression. In addition, the patient was started on piperacillin-tazobactam, valacyclovir, and fluconazole empirically for bacterial, viral, and fungal prophylaxis, respectively. Three weeks postoperatively, gastroenterology was consulted for multiple episodes of melena and hematochezia causing acute blood loss anemia, requiring several transfusions of packed red blood cells. The patient's upper endoscopy was unrevealing for a source of bleeding. Colonoscopy showed a large, inflammatory mass with cratering ulceration approximately 25 cm from the anal verge (Figure 1). Although no active bleeding was identified, nor any intervention performed, the mucosa was extremely friable and undoubtedly the source of the patient's hematochezia. Biopsy of the mass showed necrotic, inflammatory debris with hyphal fungal elements consistent with mucormycosis (Figure 2). There was no evidence of rhino-orbital disease nor diabetic ketoacidosis. On thoracic computed tomography, the patient was found to have bilateral airspace opacities consistent with aspiration pneumonia and a small right-sided pleural effusion. Pleural fluid studies and respiratory cultures were unremarkable for fungi and only grew *Klebsiella pneumoniae*. Bacterial and fungal blood cultures were unremarkable. The patient was subsequently started on acyclovir and amphotericin after an infectious disease consultation.

**Figure 3.**
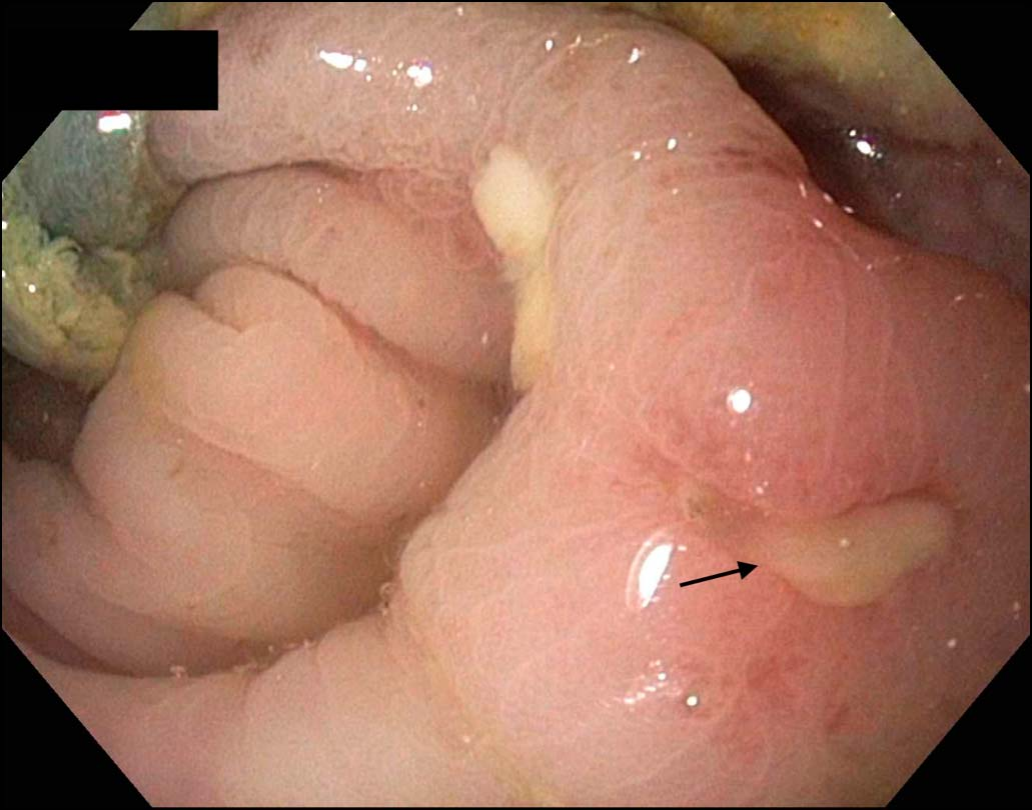
Flexible sigmoidoscopy shows a large clean based ulcer (arrow) in the sigmoid colon.

Despite therapy, the patient's hematochezia persisted. Repeat flexible sigmoidoscopy 1 week later showed clean-based ulcers; however, visualization was extremely limited because of adherent fecal material, and no endoscopic intervention was performed (Figure 3). A subsequent abdominal computed tomography scan showed a chronic walled-off perforation of the sigmoid colon. The patient subsequently underwent sigmoid hemicolectomy with primary Baker anastomosis. Surgical pathology also showed necrotic inflammatory debris with similar hyphal elements consistent with mucormycosis. Postoperatively, the patient developed an anastomotic leak and underwent abdominal washout with formation of a diverting colostomy. Given poor nutritional status and ongoing sepsis, the patient's surgical wounds could not properly heal. Subsequent wound swabs and intra-abdominal abscess cultures showed polymicrobial infection and persistent hyphal elements. Owing to significant abdominal infection with progressive septic shock complicated by multisystem organ failure, the patient experienced cardiac arrest and ultimately died 3 months after liver transplant.

## DISCUSSION

This case uniquely describes the potential morbidity and mortality of an unlikely pathogen manifesting in an extremely rare manner. Because solid organ transplant has become increasingly common over the past several decades, the incidence of systemic mycoses has risen as well. Although the exact figure is difficult to measure and likely under-represented because of the lack of formal reporting systems, the incidence of fungal infections in critically ill patients has been estimated at up to 10%.^4^ Of the invasive fungal infections, *Candida* and *Aspergillus* species are most commonly implicated. Gastrointestinal zygomycosis is exceedingly rare and is estimated to have a mortality of up to 50% of cases.^5^ This case adds to a growing body of literature that highlights the morbidity and mortality of mucormycosis infection of the gastrointestinal tract.

This case also raises many questions about the degree of immunosuppression that is appropriate post-transplant. Before liver transplant, the patient was functional, able to complete instrumental activities of daily living, and did not meet the frailty criteria. The patient had no underlying diabetes, history of immunosuppression, or history of fungal infections. After transplant, the patient received no special induction therapy for immunosuppression and was managed per hospital protocol. Despite a briefly elevated tacrolimus level, there was never any suspicion that he was inappropriately immunosuppressed because he was never leukopenic. Furthermore, once the diagnosis of mucormycosis was confirmed, mycophenolate was held and tacrolimus with low dose prednisone was continued to prevent acute rejection. Thus, the precise mechanism by which the patient developed such florid infection is unknown.

Given the infrequency of gastrointestinal mucormycosis, efficacious treatment modalities have not been extensively studied. Another case of less invasive colonic mucormycosis was successfully treated with 3 weeks of amphotericin with complete resolution.^6^ Other reviews have suggested the use of triazoles including isavuconazole and posaconazole for fungal eradication.^7^ Nonetheless, treatment drug, dose, and duration remain unclear and are often limited by toxic side effects of the aforementioned medications. In addition, surgical intervention is recommended when feasible in the case of localized infection.^7^ This patient's case also introduces the potential need for intraperitoneal treatment as a possible curative modality. Because the patient was kept on intravenous amphotericin for most his hospital course with minimal improvement, intraperitoneal wound lavage with amphotericin was used several weeks later. Despite a multimodal treatment strategy, treatment efficacy was most likely limited by the patient's multisystem organ failure, severely immunocompromised and nutritionally deplete state, and eventual polymicrobial infection. Further research is needed *in vivo* to delineate successful treatment regimens for this invasive, pathogenic microbe.

## DISCLOSURES

Author contributions: P. Aggarwal and H. Patel wrote the manuscript and reviewed the literature. L. Gonzalez, L. Brown, and K. Speeg revised the manuscript for intellectual content. A. Agarwal provided histopathology images. K. is the manuscript guarantor.

Financial disclosure: None to report.

Informed consent was obtained for this case report.
